# Misplaced Golgi Elements Produce Randomly Oriented Microtubules and Aberrant Cortical Arrays of Microtubules in Dystrophic Skeletal Muscle Fibers

**DOI:** 10.3389/fcell.2019.00176

**Published:** 2019-09-18

**Authors:** Sarah Oddoux, Davide Randazzo, Aster Kenea, Bruno Alonso, Kristien J. M. Zaal, Evelyn Ralston

**Affiliations:** Light Imaging Section, Office of Science and Technology, National Institute of Arthritis and Musculoskeletal and Skin Diseases, National Institutes of Health, Bethesda, MD, United States

**Keywords:** muscle, *mdx*, Golgi, microtubules, ERES, ERGIC, MTOC, tubb6

## Abstract

Differentiated mammalian cells and tissues, such as skeletal muscle fibers, acquire an organization of Golgi complex and microtubules profoundly different from that in proliferating cells and still poorly understood. In adult rodent skeletal muscle, the multinucleated muscle fibers have hundreds of Golgi elements (GE), small stacks of cisternae that serve as microtubule-organizing centers. We are interested in the role of the GE in organizing a peculiar grid of microtubules located in the fiber cortex, against the sarcolemma. Modifications of this grid in the *mdx* mouse model of Duchenne muscular dystrophy have led to identifying dystrophin, the protein missing in both human disease and mouse model, as a microtubule guide. Compared to wild-type (WT), *mdx* microtubules are disordered and more dense and they have been linked to the dystrophic pathology. GE themselves are disordered in *mdx*. Here, to identify the causes of GE and microtubule alterations in the *mdx* muscle, we follow GFP-tagged microtubule markers in live *mdx* fibers and investigate the recovery of GE and microtubules after treatment with nocodazole. We find that *mdx* microtubules grow 10% faster but in 30% shorter bouts and that they begin to form a tangled network, rather than an orthogonal grid, right after nucleation from GE. Strikingly, a large fraction of microtubules in *mdx* muscle fibers seem to dissociate from GE after nucleation. Moreover, we report that *mdx* GE are mispositioned and increased in number and size. These results were replicated in WT fibers overexpressing the beta-tubulin tubb6, which is elevated in Duchenne muscular dystrophy, in *mdx* and in regenerating muscle. Finally, we examine the association of GE with ER exit sites and ER-to-Golgi intermediate compartment, which starts during muscle differentiation, and find it persisting in *mdx* and tubb6 overexpressing fibers. We conclude that GE are full, small, Golgi complexes anchored, and positioned through ER Exit Sites. We propose a model in which GE mispositioning, together with the absence of microtubule guidance due to the lack of dystrophin, determines the differences in GE and microtubule organization between WT and *mdx* muscle fibers.

## Introduction

It has long been known that Golgi complex (GC) and microtubules are tightly interwoven (Burkhardt, 1998). However, there is a large gap between their organization in proliferating cells and in differentiated tissues (Yadav and Linstedt, 2011). In proliferating cells, the main features of GC and microtubule organization are now understood at a molecular and mechanistic level (Wei and Seemann, 2017). Briefly, microtubules form a single radial array growing from the centrosome (or microtubule-organizing center, MTOC) to the cell membrane. A single GC is dynamically kept near the centrosome through the action of minus-end directed microtubule motors. This organization is well-suited to the needs of cell division in which microtubules play an essential role. Some proliferating cells, however, show two distinct sets of microtubules: one radial array nucleating from the centrosome and one directional array originating from the GC itself (Efimov et al., 2007; Rios, 2014) and involved in functions such as cell migration (Miller et al., 2009).

When cells leave the mitotic cycle and differentiate, microtubule, and GC organization are profoundly changed to support the architecture and function of the developing tissue. Different cell types such as those found in muscle, plants, and brain, adopt very different, complex, subcellular organizations (Oddoux et al., 2013; Chen et al., 2016; Mikhaylova et al., 2016). Few of these organizations are understood at a mechanistic level and even at a descriptive level there remains a lot to understand. This is the case in myogenesis (skeletal muscle development). Extensive reorganizations take place during differentiation, which can be studied in cell culture. However, muscle cell cultures do not replicate the subsequent changes that take place during muscle maturation and regeneration and are key to the role of GC and microtubules in muscle diseases.

Undifferentiated muscle cells (myoblasts) have the typical organization of proliferating cells described above. As muscle-specific transcription factors become expressed, myoblasts stop dividing. Their MTOC redistributes to the nuclear membrane (Tassin et al., 1985a). Microtubule nucleation and GC follow (Tassin et al., 1985b). The single GC is replaced by numerous smaller Golgi elements (GE) and the radial microtubule array replaced by mostly parallel microtubules, which contribute to elongation of the cell shape (Zaal et al., 2011). GE are small stacks of cisternae. They are positioned next to ER exit sites (ERES), as are GC fragments of proliferating cells treated with drugs that depolymerize microtubules (Cole et al., 1996; Lu et al., 2001). GE and ERES, from this stage on, are not distinct organelles positioned close by but are single secretory units formed of two parts. The next important change in cellular architecture is the fusion of the differentiated myoblasts (also known as myocytes) to form multinucleated myotubes. Myotubes in muscle cell cultures can reach hundreds of microns in length, with tens of nuclei distributed from end to end in a seamless cytoplasm.

*In vivo*, myotubes mature into muscle fibers, arguably the largest mammalian cells (with radii up to 100 μm). Innervation is an essential step in the maturation of myotubes into muscle fibers. Once innervated, muscle fibers are exposed to stimulation by patterned activity, which regulates fiber contraction, speed, metabolism (Schiaffino et al., 2015), and subcellular organization, including that of microtubules and GE (Ralston et al., 2001). Muscle fibers are divided into different types, related to their activity (fast, slow, and intermediate) but they are plastic: changes in contractile activity, experimental or due to disease, remodel muscle fibers (Dowling et al., 2016).

When considering the radial organization of muscle fibers, two domains can be distinguished: core and cortical areas. Their main distinguishing feature is the concentration, in the core, of the contractile myofibrils and of the organelles involved in excitation-contraction; the cortex is a thin lining that resembles the cytoplasm of non-muscle cells. The fiber cortex houses the nuclei which are distributed evenly, radially and longitudinally. Mitochondria, microtubules and GE are found in both cortex and core, but their organization is different in each domain and depends on fiber type (Ralston et al., 1999). In particular, GE and microtubules form a unique orthogonal grid in the fiber cortex of all except slow-twitch fibers, i.e., in most muscles of the mouse. This grid is composed of single and bundled microtubules, with GE positioned at microtubule intersections. The microtubule grid periodicity matches that of the contractile proteins; longitudinal microtubules are aligned with the axis of the muscle fiber and transverse microtubules are aligned with Z-disks (Kaisto and Metsikkö, 2003).

To find out whether this microtubule grid is dynamic and to resolve the “chicken-egg” question of whether microtubules position GE or GE position microtubules, we investigated them both in live muscle fibers expressing fluorescently tagged markers (Oddoux et al., 2013). These experiments were done on collagenase-dissociated fibers of the mouse *flexor digitorum brevis* (FDB) muscle and were further validated by intravital recordings (Oddoux et al., 2013). We found out that muscle GE are static and nucleate dynamic microtubules that grow along stable microtubules, all together forming a permanent grid superimposed on the orthogonal network formed by dystrophin (Prins et al., 2009).

Dystrophin is the protein that is absent in Duchenne muscular dystrophy (DMD), an X-linked fatal muscle disease, and in the *mdx* mouse, a DMD animal model (Bulfield et al., 1984; Hoffman et al., 1987). Several observations led to the proposal that microtubule-dystrophin interactions play a role not only in muscle fiber organization but also in the pathology of DMD. First, microtubules and GE of *mdx* muscles were found to be disorganized and rescued by expression of a micro-dystrophin construct (Percival et al., 2007). Importantly, dystrophin was shown to bind microtubules (Prins et al., 2009; Belanto et al., 2014) and to be positioned along them (Prins et al., 2009). Microtubules have also been identified as part of the dystrophin complexome (Murphy and Ohlendieck, 2016). Dystrophin is a constituent of the costameres (Ervasti, 2003), protein assemblies of the muscle cortex that form periodic links between sarcolemma and myofibrils at the level of the Z-disks, thus perfectly positioned to interact with cortical microtubules. Finally, microtubules were implicated in dystrophinopathy: the production of reactive oxygen species (ROS) and other hallmarks of DMD, upon experimental stretching of *mdx* muscle fibers *ex vivo*, could be prevented by depolymerizing microtubules (Khairallah et al., 2012; Kerr et al., 2015).

However, recent work has made matters more complicated. First, a grid-like microtubule organization and normal physiology was restored in a transgenic *mdx* mouse line expressing a dystrophin construct lacking the microtubule-binding domain (Belanto et al., 2016). Then, microtubule organization was rescued in an *mdx* mouse line following elimination of ROS production without restoration of dystrophin expression (Loehr et al., 2018). The same work showed that fibrosis plays a larger role than microtubules in *mdx* pathology. Finally, we found out that experimentally overexpressing the β-tubulin tubb6, which is highly elevated in DMD and *mdx* muscle, destroys the microtubule grid of wild-type (WT) mouse muscles (Randazzo et al., 2019). Conversely, knocking down tubb6 in *mdx* muscle fibers improved their microtubule organization (Randazzo et al., 2019). Thus, dystrophin seemed neither necessary nor sufficient to organize muscle microtubules. Additionally, it is known that the mere presence of dystrophin in a muscle fiber is not sufficient to ensure its positioning in the costameres. Instead, dystrophin binding to the costameres depends on a cascade of proteins including ankyrin-B, spectrin, dynactin-4, and, amazingly, microtubules (Ayalon et al., 2008, 2011). Furthermore, dystrophin positioning, like that of microtubules and GE, depends on muscle activity (Bezakova and Lømo, 2001).

**Figure 1:**
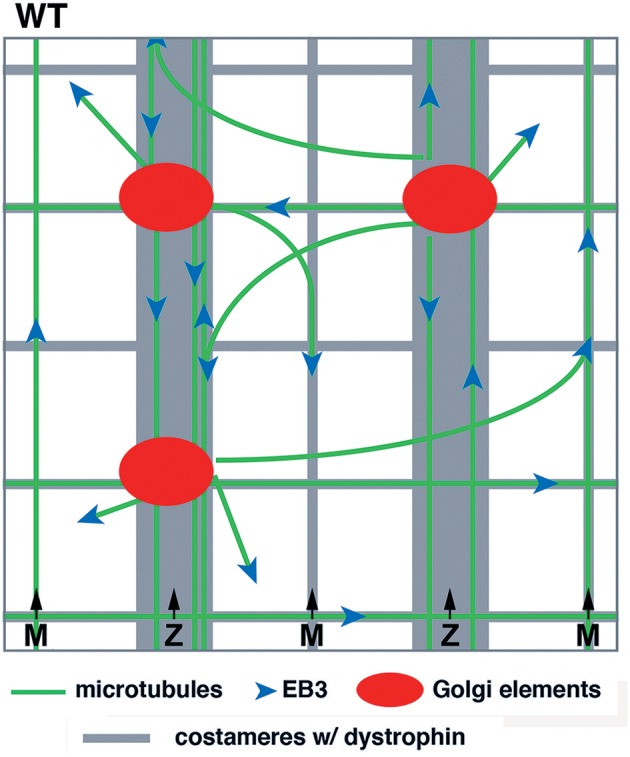
Model of GE and microtubule organization in the cortex of WT muscle fibers. GE and microtubules both form an orthogonal grid that is aligned with dystrophin. Dystrophin is part of the costameres (muscle “ribs”) in the transverse direction and also forms longitudinal lines. Dynamic microtubules, with the protein EB3 marking their growing plus-end, are nucleated on the GE and grow bidirectionally mostly along dystrophin tracks. GE are static and the whole grid is stable over time.

Our previous work with WT muscle fibers led to a model, schematically represented in Figure 1. Several features were important: first, GE themselves formed a grid and were anchored along the transverse and longitudinal components of dystrophin; second, microtubules grew from the GE in a limited range of directions, mostly transverse and longitudinal; third, dystrophin was available for guidance. Here, we investigate the consequences of the absence of guidance by dystrophin in *mdx* fibers. We follow microtubule dynamics in live and fixed FDB fibers of the *mdx* mouse and follow recovery of the microtubule network from nocodazole depolymerization. We find that the differences in microtubule directionality and density can be observed at the earliest stage of microtubule growth but that the main features of the WT network, i.e., a dynamic though permanent microtubule network are maintained in *mdx* fibers. Furthermore, GE are associated with ERES in *mdx* as they are in WT fibers. We conclude that mispositioning of muscle GE, which are full, though small, Golgi complexes, and the random orientation of growing microtubules cause microtubule disorder in *mdx* muscle.

## Results

### GFP-Tagged Microtubule Markers Visualized in Live WT and *mdx* Muscle Fibers Highlight Directionality and Density Differences

**Figure 2:**
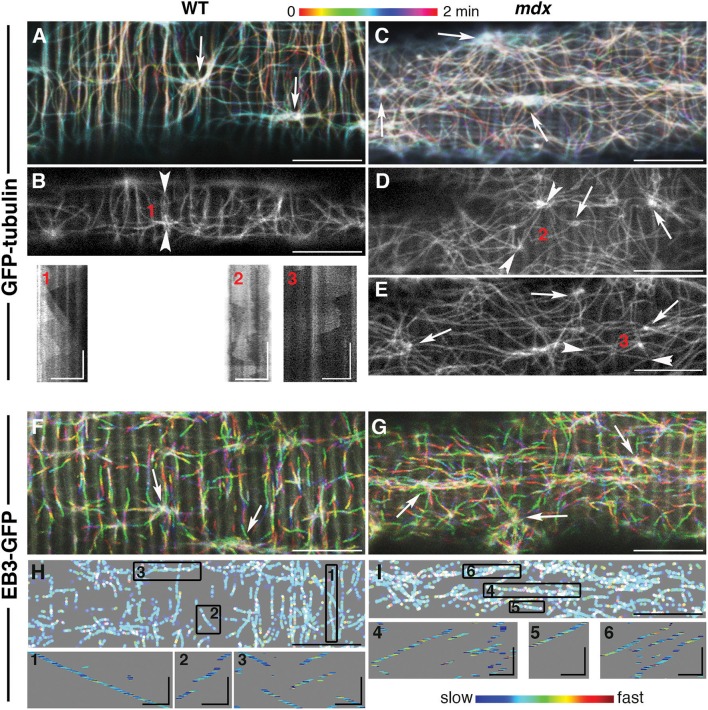
While *mdx* and WT muscle microtubules differ in their pattern and density, both have dynamic but stable microtubule networks nucleated from stationary MTOC. Muscle fibers from the *flexor digitorum brevis* muscle expressing GFP-tubulin or EB3-GFP, as indicated on the left side, were imaged at the rate of 93 f/min with a 63x N.A. 1.4 objective on a Leica SP5 confocal microscope fitted with a heating stage and objective heater at 37 deg. **(A,C,F,G)** show projections of 2 min recordings. These recordings are in the Supplementary Videos 1–7 and correspond to **(A–G)**. The long axes of the muscle fibers are parallel with the long side of the images. The projections were color-coded to indicate time, as shown by the bar above the images. The main differences between WT and *mdx* microtubules are their directionality and density. **(A,F)** show many transverse microtubules while panels **(C,G)** show very few, if any. Because microtubules form bundles and grow along one another, GFP-tubulin recordings show little color, unless a new track is formed or a microtubule shrinks. In EB3 recordings, only growing microtubules are seen since EB3 detaches from shrinking microtubules. White tracks (merging of rainbow colors) are those present through the recordings. Arrowhead pairs in images **(B,D,E)** bookend lines selected to draw kymographs (1–3) of GFP-tubulin sequences. A kymograph is a time-distance graph obtained by repetition of the same line for each time-point. Kymograph 1 reflects successive production of microtubules that grow along the line at steady velocity; kymographs 2–3 reflect the presence of unstable microtubules both along and across the kymograph line. For EB3-GFP recordings, the color coding in panels H and I represents the relative instantaneous velocity from blue for the slowest to red for the fastest. Kymographs of selected tracks (boxes 1–6) show that EB3 comets do not progress at a constant speed but speed up and slow down abruptly but EB3-GFP growth velocity is independent of the growth direction. These projections were thresholded and binarized to suppress the background striations visible in F, G and inherent to EB3-GFP expression. Bars: **(A–H)**: 10 μm; **(I)**: 2 μm. In the kymographs, vertical bars: 30 s, GFP-tubulin horizontal bars: 5 μm; EB3-GFP horizontal bars: 2 μm.

To compare the dynamic features of *mdx* GE and microtubules to those of WT, we followed fluorescent markers of α-tubulin (GFP-tubulin) and of the plus-end microtubule protein EB3 (EB3-GFP) in live FDB fibers of the two genotypes (Figure 2) as previously done for WT muscle fibers only (Oddoux et al., 2013). The video recordings (found in the Supplementary Material) are presented here as color-coded projections in which color serves as an indication of time (Figures 2A,C,F,G represent Supplementary Videos 1, 3, 6, 7). Panels B, D, and E present single images of GFP-tubulin recordings (Supplementary Videos 2, 4, 5).

An even superficial examination of the panels reveals *mdx* vs. WT differences in microtubule directionality: WT panels show numerous transverse (vertical) microtubules lacking from *mdx* panels. These differences were confirmed by analysis with the TeDT microtubule directionality software (Liu and Ralston, 2014; Supplementary Figure 2A).

The color coding of the projections also gives a snapshot of microtubule dynamics: stationary microtubules appear white or lightly colored (Figures 2A,C), while projection of the growing plus-ends forms tracks of successive colors (Figures 2F,G). In this respect, *mdx* recordings look similar to WT ones, indicating that, in both, dynamic microtubules grow along stable microtubules. Kymographs (boxes 1–3) give a different visual presentation of the complexity of microtubule dynamics in *mdx*. To obtain these, a line was drawn along a microtubule track (between two arrowheads in Figures 2B,D,E) and plotted as a stack with the line repeated at each time-point (see methods). The WT kymograph, with successive triangles of similar slopes, indicates repetitive growth of microtubules along the kymograph line, from a common point of origin and with constant velocity. The *mdx* kymographs, in contrast, reflect longer or shorter-lived and random-oriented microtubules that do not precisely follow the central line. In addition, the density of *mdx* microtubules is higher than that of WT.

The interpretation of the visual differences in the kymographs is supported by quantitation of microtubule growth parameters (Table 1). Two parameters differ significantly: *mdx* microtubules grow significantly faster than WT ones by ~10% and their growth duration is shorter by ~30%.

**Table 1:** Microtubule dynamic parameters (α-tubulin-GFP), *mdx* vs. WT.

	**WT**	***mdx***	***p***
Growth speed (μm/min)	7.8 ± 0.2 (*N* = 149)	8.6 ± 0.3 (*N* = 123)	*0.0003
Growth distance (μm)	1.3 ± 0.1 (*N* = 149)	1.3 ± 0.1 (*N* = 122)	>0.05
Growth duration (s)	11.9 ± 1.0 (*N* = 149)	8.8 ± 0.6 (*N* = 122)	*0.0022
Shrinking speed (μm/min)	14.5 ± 0.7 (*N* = 107)	14.0 ± 0.6 (*N* = 87)	>0.05
Shrinking distance (μm)	1.7 ± 0.2 (*N* = 108)	1.5 ± 0.2 (*N* = 91)	>0.05
Shrinking duration (s)	6.7 ± 0.6 (*N* = 108)	5.8 ± 0.4 (*N* = 90)	>0.05
Pause duration (s)	7.8 ± 0.7 (*N* = 108)	7.8 ± 0.8 (*N* = 90)	>0.05

Movement analysis was done in ImageJ as described in Methods. Image sequences were taken from 3 WT and 3 mdx FDB muscles, each one from a different animal. For each one, 15 fibers were imaged for 2 min at 0.65 frames/ms. For WT fibers, a total of 42 microtubules were included in the calculations; for mdx fibers, a total of 47 microtubules. The stars (*) tag the p values that indicate statistical significance.

In all projections, MTOC (arrows in Figures 2A–G) appear as lighter colored points shown in videos and kymographs to be static. Watching them in loops, we started noticing differences between WT and *mdx* recordings. WT MTOC are distant enough from one another to not interfere. Microtubules grow periodically in only a few directions, mainly along or at 90 degrees off the fiber axis. In *mdx* recordings, in contrast, new microtubule growth appears stochastic in both direction and timing. Microtubules originating elsewhere often cross the area under observation. Examples of one WT and one *mdx* MTOC and surroundings are shown in projection in Supplementary Figure 1 and in Supplementary Video 8 (WT) and Supplementary Video 9 (*mdx*), respectively.

Thus, the presence of dystrophin seems to be necessary for spatial and temporal organization of microtubules.

### Differences Between *mdx* and WT Golgi-Microtubule Patterns Manifest Early on During Microtubule Recovery From Nocodazole-Induced Depolymerization

**Figure 3:**
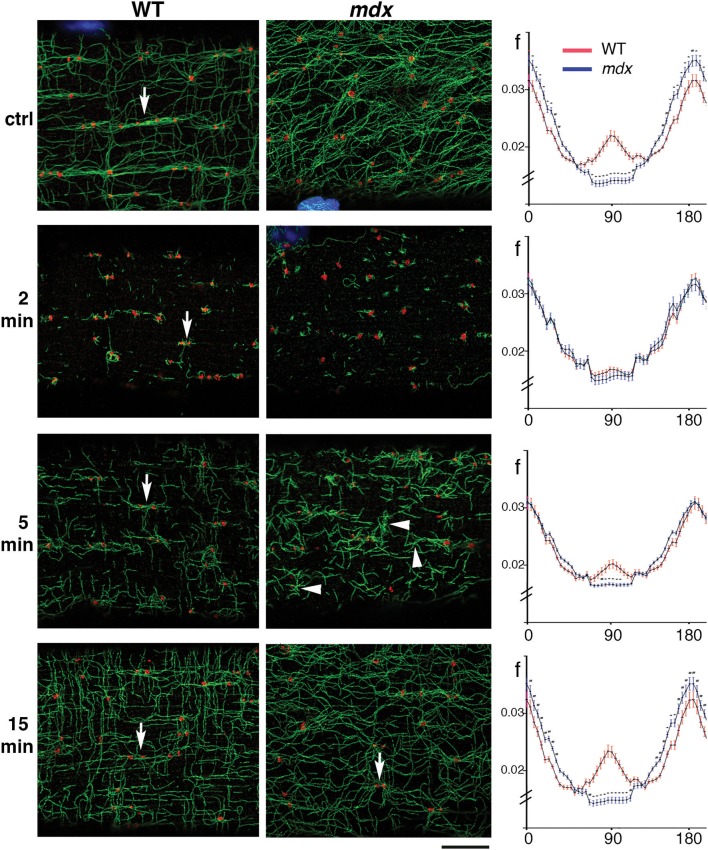
During recovery from microtubule depolymerization, differences between *mdx* and WT fibers are established before a connected network can be seen. FDB fibers from WT and *mdx* muscles were treated with nocodazole (see Methods) to obtain complete depolymerization of dynamic microtubules. Nocodazole was then washed out and fibers fixed and stained after 2, 5, and 15 min of recovery. Panels of the WT and *mdx* columns show single confocal images of immunofluorescence staining for GE (GM130, red) and dynamic microtubules (tyrosinated tubulin, green) at steady state (ctrl), and after the indicated time of nocodazole washout. Impressively, the original network organization is almost recovered in 15 min. At 2 min, GE appear as mostly independent seeds with short microtubules. After 5 min, microtubules form asters (groups of microtubules with a common point of origin) and start to connect. Some of the asters, especially in *mdx* samples (arrowheads) do not seem to be associated with a GE (see text). In WT samples, control fibers show groups of GE along microtubule bundles (arrow). After 2 and 5 min recovery, only pairs of GE (arrows) are seen. At 15 min they appear to be connecting. The directionality of the MTs at each stage was analyzed with the TeDT software (right side column). The curves represent the normalized frequency of the angles that microtubules form with the fiber axis, with 0°/180° corresponding to the longitudinal fiber axis. In the control, significant differences exist along the whole range of angles: WT microtubules have a peak at 90° that is absent in *mdx* microtubules and are also more polarized into longitudinal and transverse microtubules while *mdx* microtubules are found in more orientations. After 2 min of nocodazole washout, the curves are very close but 5 min suffice to make them significantly different. After 15 min, the distributions and density of WT and *mdx* MTs are similar to those in the steady state (*p < 0.0001). Directionality measurements were done on two areas per fiber with 18 *mdx* and 17 WT steady-state fibers, 16 *mdx* and 14 WT fibers for 2 min, 15 *mdx* and 15 WT fibers for 5 min and 20 *mdx* and 20 WT fibers for 15 min time points. The fibers were representative of three independent experiments. Bar: 8 μm.

To verify that GE are the MTOC of *mdx* as they are those of WT microtubules, and to find out more about microtubule orientation at early growth stages, we decided to disassemble the muscle fiber microtubule network and to watch GE and microtubules during recovery. After treatment for 4 h with the drug nocodazole to depolymerize microtubules (see methods), microtubules were rare, with only stable, nocodazole-resistant microtubules left (not shown). Nocodazole was washed out and fibers were left to recover for 2, 5, and 15 min. Untreated and recovered samples were then stained for the cis-Golgi marker GM130 and for tyrosinated α-tubulin, a marker of dynamic microtubules. Representative images from three independent experiments as well as directionality measurements are shown in Figure 3. After 2 min of recovery, GE were surrounded by short microtubules in both genotypes but were positioned differently. WT GE often formed doublets (arrows) aligned with the axis of the fibers, while *mdx* GE formed fewer doublets and were unaligned. An orthogonal grid could be superimposed on an image of WT but not of *mdx* GE. Most microtubules were shorter than 1 μm and surrounded by GE but some appeared to be detached from GE. At this stage there were no significant differences in the directionality plots which did not show any preferential directionality. Between 2 and 5 min, microtubules grew and established significant differences in density and directionality between *mdx* and WT samples. A majority of WT microtubules remained connected to GE but *mdx* microtubules filled out the space. Microtubule clusters apparently without GE, some shaped as asters, were present (Figure 3 arrowheads and Supplementary Figure 2A). We counted such microtubule clusters in confocal images from three independent experiments. In WT fibers, 28% (*n* = 126) had no GM130 staining. In *mdx* fibers this proportion increased to 55% (*n* = 162). The difference between *mdx* and WT was highly significant (*p* < 0.001). At 15 min recovery, a full network was reorganized that appeared to link all microtubules. The muscle fiber surface occupied by microtubules and by nucleating GE differed between *mdx* and WT as did the ratio of microtubules to GE number (Supplementary Figure 2B). Thus, GE remain the main MTOC in *mdx* muscle fibers (besides nuclei) but they are positioned in a disordered way. The long treatment in nocodazole did not cause further fragmentation of GE as would be the case for a classical GC. It did not cause their fusion either. Thus, GE remain anchored in the absence of dystrophin but the anchoring points are not positioned along a grid. Anchoring and positioning of GE may be regulated by distinct mechanisms. Furthermore, the presence in *mdx* fibers of an increased proportion of microtubules not clearly associated with GE likely contributes to the higher density and the disorder of the *mdx* microtubule network.

### Nuclei-Originating Microtubules Are Stabilized in *mdx* Fibers

**Figure 4:**
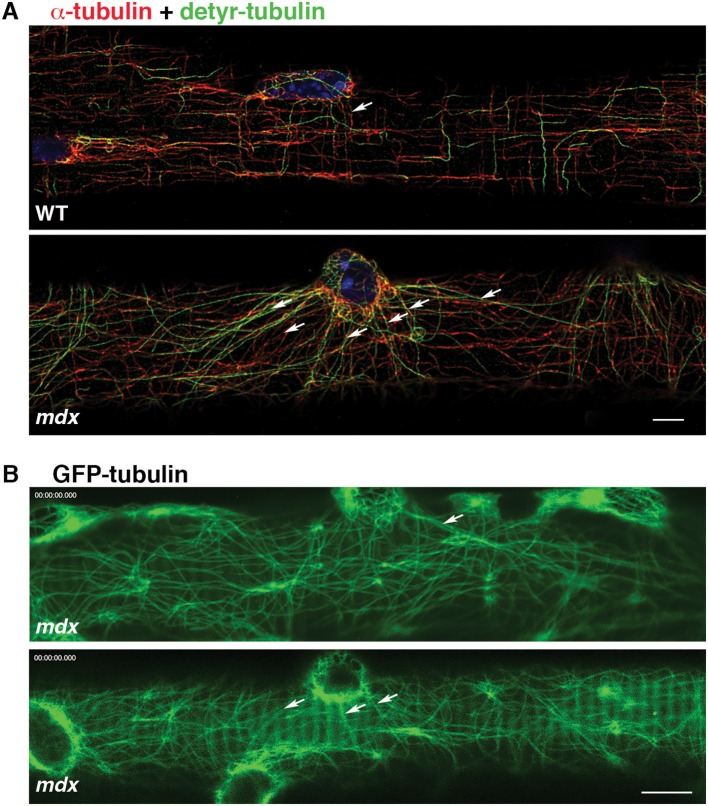
Microtubules nucleated from *mdx* nuclear membranes are long and stable. Cortical microtubules include some that are nucleated on the nuclear membrane (arrows). In WT fibers they are part of the microtubule grid but in *mdx* fibers they are distinct. **(A)** Immunofluorescence of FDB fibers for α-tubulin (red), detyrosinated tubulin (green) and nuclei (blue); **(B)** Single frames of GFP-tubulin recordings. Bars: **(A)** 5 μm; **(B)** 7.5 μm.

In addition to GE-nucleated microtubules, the cortical area of muscle fibers has microtubules originating from nuclear membranes. In WT fibers, such microtubules join the grid and do not stand out (Oddoux et al., 2013). However, in *mdx* FDB fibers stained for dynamic and stable microtubules we noticed long microtubule stretches radiating from nuclear membranes (Figure 4A, red) and positive for detyrosinated tubulin (Figure 4A, green), which marks stable microtubules. Similar patterns were also found in GFP-tubulin recordings of *mdx* fibers (Figure 4B). Thus, microtubules originating from nuclear membranes are stabilized and reach longer distances in *mdx* than WT fibers, forming a distinct set of microtubules, often linking neighboring nuclei. Nuclear dynamics are known to be altered in *mdx* fibers (Iyer et al., 2017).

### GE Positioning and Size Distribution Are Altered in Both *mdx* and tubb6 OE Fibers

**Figure 5:**
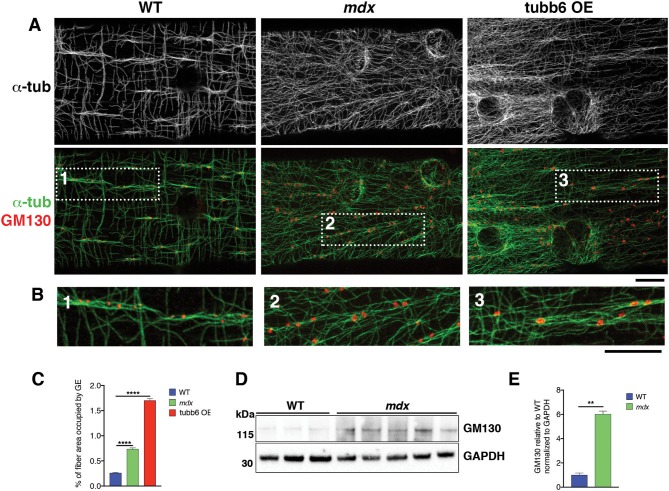
GE differ in organization, number and surface in *mdx* and tubb6 OE compared to WT fibers. **(A)** Single confocal images of FDB fibers stained for GM130 (red) and α-tubulin (green or black and white in the first row) show lines of GE along microtubule bundles, highlighted in dotted frames and shown in **(B)** at higher magnification. In WT fibers, GE are mostly paired or grouped along longitudinal microtubule bundles. WT GE are smaller than those of *mdx* or tubb6 OE fibers. In *mdx* or tubb6 OE fibers, microtubule bundles, when present, are at an angle with the axis of the fiber. **(C)**. The % of fiber surface covered by GE is increased in *mdx* and tubb6 OE fibers. The number of GE analyzed was 1,269 for WT, 1,485 for *mdx*, and 4,185 for tubb6 OE. Images were collected from three independent experiments; (****p < 0.0001). **(D,E)**. Immunoblotting of *gastrocnemius* muscle extracts for GM130 shows a 6-fold increase in *mdx* muscle extracts (**p < 0.01), confirming an increase in GE components. Bars: 10 μm.

If GE mispositioning is part of the *mdx* microtubule disorder, we expect other conditions that cause a similar microtubule disorder to involve GE alterations as well. We thus tested the effect on GE of overexpression of the β-tubulin tubb6 in WT fibers, a condition that mimics several aspects of *mdx* dystrophinopathy (Randazzo et al., 2019). After staining WT, *mdx*, and tubb6 OE fibers for the cis-Golgi protein GM130 and for microtubules we noticed differences in GE positioning (Figure 5A). In WT fibers, most GE (79%) are found in pairs or small groups, along microtubule bundles (see Figure 5A and enlarged details in Figure 5B) that are parallel to the fiber axis. Such groups are less frequent in *mdx* (28%) whose GE are found along bundled or single microtubules that have various orientations at an angle with the fiber axis. Tubb6 OE fibers showed similarly dense and disordered microtubules with an intermediate proportion of grouped GE (50%) and occasional longitudinal bundles. Quantitation is presented in Table 2. We also noted that the size of GE is more variable in *mdx* and tubb6 OE WT fibers than in WT fibers, with some of them quite larger. This is not related to a change in the whole fiber: the proportion of fiber surface occupied by GE in muscle fibers is significantly increased for *mdx* and tubb6 OE fibers (Figure 5C). Immunoblots of WT and *mdx gastrocnemius* muscle extracts for GM130 also showed a significant increase of the GE marker (Figures 5D,E). Golgi distribution is thus disordered in tubb6 OE fibers as in *mdx* fibers and the surface of GE in both *mdx* and tubb6 OE fibers is significantly increased compared to WT.

**Table 2:** Quantitation of GE and of their association with microtubule bundles in WT, *mdx*, and tubb6-overexpressing muscle fibers.

	**GE organization**	**numbers**	**in %**
WT	Total (3 images)	147	100
	With bundles	116	79
	With nuclei	5	3
	With transverse Microtubules	1	0
*mdx*	Total (5 images)	444	100
	With bundles	123	28
	With nuclei	87	20
	With transverse microtubules	1	0
tubb6-overexpressing	Total (3 images)	508	100
	With bundles	252	50
	With nuclei	70	14
	With transverse microtubules	2	0

*Confocal images of GM130 and α-tubulin staining were counted in ImageJ for the total number of GE and manually for the number of those GE associated with microtubule bundles, with nuclei, and with transverse microtubules. Remaining GE are single and associated with unbundled microtubules*.

### WT GE Aligned in Rows Do Not Share a Homogenous *cis-trans* Polarity

**Figure 6:**
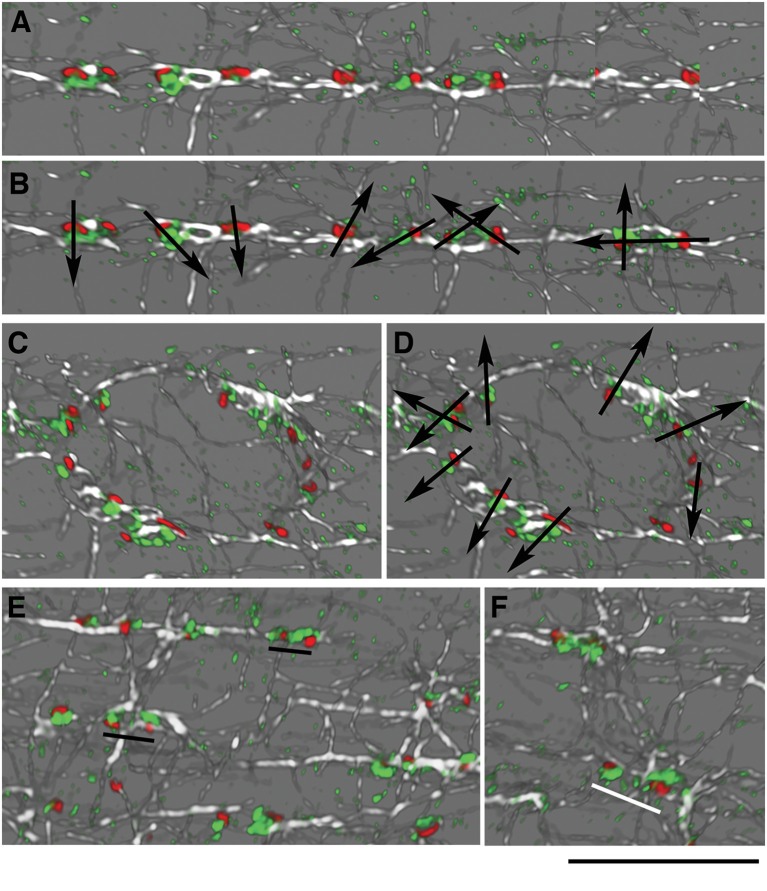
GE are not oriented in concert along microtubules in WT fibers. Concerted orientation of GE could explain the directionality of microtubule nucleation. To determine the *cis-trans* orientation of GE in WT fibers, these were stained for GM130 (*cis*-Golgi, red), GLUT4 (*trans*-Golgi, green), and α-tubulin (white). Imaging was done at increased resolution with Lightning on the Leica SP8 confocal and representation in 3D in the LASX software. **(A,B)** *cis-trans* polarity (represented by arrows in **B**) differs for all GE, even found along common microtubules. **(C,D)** GE along a nuclear membrane showed the expected *cis-trans* polarity, with the cis-Golgi on the nuclear membrane side. **(E,F)** Some pairs of GE have the same polarity (**E**, black underlining) while others look as if rotated 180 deg (**F**, white underlining). Bar: 10 μm.

In the cartoon of Figure 1, GE are represented as small blobs, as we view them in light microscopy with a single marker. Staining of muscle fibers with markers of both *cis*- and *trans*-Golgi cisternae, however, resolves cis-trans polarity of the GE (Ploug et al., 1998). Around nuclei, GE have a uniform polarity with the *cis-trans* axis pointing away from the nuclear membrane. The orientation of the GE along costameres could impose a directionality to the microtubules they nucleate. If this were the case, we would expect a row of GE along WT costameres to have a uniform *cis-trans* polarity, but we would expect *mdx* and tubb6 OE GE to be randomly oriented. To evaluate this hypothesis, we needed a *trans*-Golgi marker but none of the *trans*- specific antibodies that we tested gave an acceptable staining of mouse muscle fibers. We then used the P-1 antibody against the muscle and fat cell-specific glucose transporter GLUT4, which accumulates in the TGN and colocalizes with TGN38 (Ploug et al., 1998). We carried out imaging in the super-resolution Lightning mode of the Leica SP8 confocal and displayed the results in 3D (Figure 6, see methods). GLUT4 is not only in the TGN, hence small puncta show up in the staining. In WT fibers, however (six images with a total of 159 GE), we found no uniform polarity along GE rows (Figure 6A), although the expected *cis-trans* polarity was present in the perinuclear GE (Figure 6B). Occasional close pairs of GE had common polarity (Figure 6C, black underlining), others did not (Figure 6C, white underlining). *Mdx* and tubb6 OE fibers showed equally uncoordinated GE orientation (data not shown). We therefore rule out alignment of GE with all *cis-trans* axes in the same direction as a source of microtubule alignment in WT muscle fibers.

### GE, ERES, and ER to Golgi Intermediate Compartment (ERGIC) Remain Juxtaposed in *mdx* and tubb6 OE Muscle Fibers and Undergo Correlated Volume Changes

**Figure 7:**
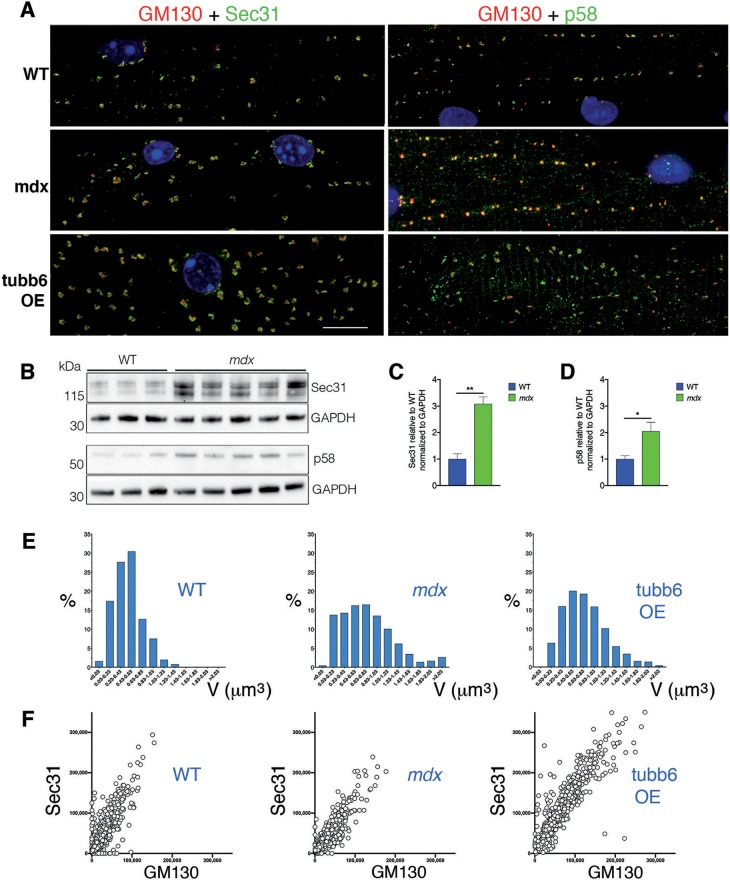
GE-ERES and GE-ERGIC assemblies in *mdx* and tubb6 OE fibers are larger than in WT fibers. **(A)** In mature FDB muscle fibers stained for GM130 (red) and Sec31 or p58 (green), GE are more numerous than in muscle cultures (Supplementary Figure 3) but smaller and tightly associated with ERES or ERGIC. The three subcellular compartments differ in number and size between WT, *mdx*, and tubb6 OE fibers. The fixation procedure for p58 differed from that for Sec31 (see methods), possibly contributing to a higher background for p58. **(B)** Immunoblots of *gastrocnemius* muscle show that there is a 3-fold significant increase of Sec31 (**p < 0.01) and a 2-fold significant increase of p58 (*p < 0.05) in *mdx* compared to WT extracts **(C,D)**. **(E)** To quantitate in 3D we used the software Imaris. We first calculated GE+ERES volumes and plotted them as a histogram showing proportion of each size to the total. **(F)** We also plotted Sec31 vs. GM130 staining intensity (in arbitrary units) measured for each GE, also in Imaris, and plotted one against the other. The number of GE included in calculations E and F was 1,052 for WT, 957 for *mdx*, and 1,013 for tubb6 OE, with *n* = 3 animals per group. Quantitation thus confirms the increase in size from WT to *mdx* and *mdx* to tubb6 OE; it also shows that the volume of GE and ERES stainings are correlated, regardless of genotype or condition. Bar: 10 μm.

The concerted reorganization that brings together Golgi and ERES during muscle differentiation (Lu et al., 2001) turns GE into dynamically tethered extensions of the ER and is thus important for their localization. We asked whether this organization is maintained in *mdx* and tubb6 OE fibers and whether ERGIC are reorganized simultaneously to ERES (Saraste and Marie, 2018). Immunostaining of differentiating C2 muscle cells for GM130 and the ERES protein Sec31 (Shugrue et al., 1999) or the ERGIC protein p58 (Lahtinen et al., 1996) shows a similar reorganization of GE and ERGIC (Supplementary Figure 3). Staining of FDB fibers (Figure 7A and Rahkila et al., 1997) reveals the tight assemblies of GE-ERES and GE-ERGIC. In *mdx* and tubb6 OE fibers they remain but are larger. ERES in each of the three samples surround GE, while p58 appears colocalized with the cis-Golgi marker, as expected. Immunoblots show both Sec31 and p58 significantly increased in *mdx* fibers (Figures 7B–D) and volume measurements in the software Imaris show different distributions and larger sizes in *mdx* and tubb6 OE fibers (Figure 7E). Finally intensity measurements show that GE and ERES staining correlate (Figure 7F). It is generally accepted that reciprocal interactions between GC and ERES determine their size, which is linked to the demand for protein transport (Hammond and Glick, 2000; Stephens, 2003; Sengupta and Linstedt, 2011; Glick, 2014).

**Figure 8:**
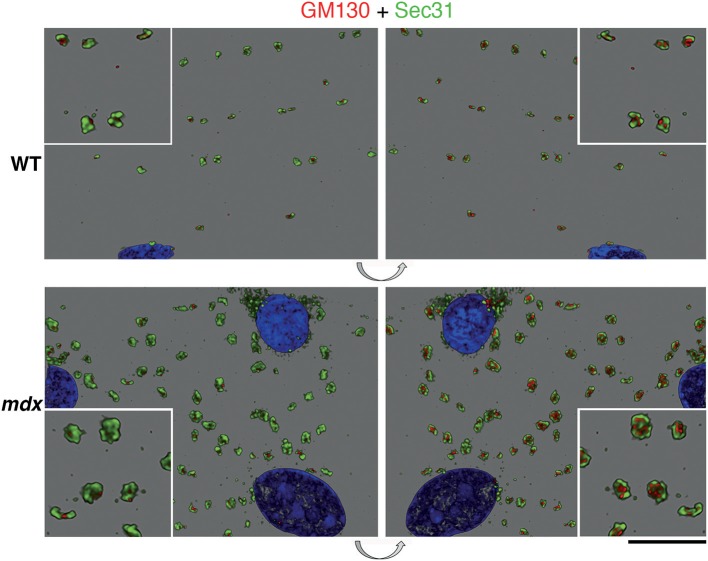
Polarity of GE vs. ERES organization and correlation of their volume. Z-stacks of confocal images showing GE (GM130 staining, red) and ERES (Sec31 staining, green) were collected at increased resolution with Lightning and represented in 3D in the LASX software. The volume difference between WT and *mdx* images is striking but in addition there is a polarization of the ERES-GE axis. In this representation the staining is opaque. The left panels show the staining as viewed from outside the fiber, the right panels, obtained by 180 deg flipping (see arrows), show the same image viewed from inside. It thus appears that the *cis*-Golgi cisternae, surrounded by Sec31, are on the cytoplasmic side. Bar: 10 μm for the main panels and 5 μm for the enlarged insets.

We then examined GE and ERES association in Lightning super-resolution and in 3D. The images revealed, unexpectedly, that GE-ERES in *mdx* and WT are polarized, with ERES on the sarcolemmal side (Figure 8), suggesting that cortical muscle GE are associated with an ER-like subsarcolemmal tubular system described by Jayasinghe et al. (2013). The association between GE and ERES is thus maintained in *mdx* and tubb6 OE fibers despite changes in their respective sizes, possibly indicating increased protein trafficking.

Finally, counting GM130's pairwise association with ERES, ERGIC and TGN markers, in WT, *mdx*, and GFP-tubb6 OE fibers (Table 3) indicated that muscle GE are all small but fully functional Golgi complexes, unlike neuronal Golgi which include Golgi outposts and an incomplete Golgi-related satellite organelle (Mikhaylova et al., 2016).

**Table 3:** GM130 puncta association with markers of ERES, ERGIC, and TGN.

**GM130-sec31 staining**	**GM130**	**Sec31**
WT	540 (7 fibers)	540
*mdx*	508 (7 fibers)	508
GFP-tubb6-overexpressing	557 (5 fibers)	557
**GM130-GLUT4 staining**	**GM130**	**GLUT4**
WT	496 (8 fibers)	496
*mdx*	527 (8 fibers)	527
GFP-tubb6-overexpressing	887 (7 fibers)	887
**GM130-p58 staining**	**GM130**	**p58**
WT	302 (4 fibers)	302
*mdx*	288 (4 fibers)	288
		

*To examine whether muscle GE all represent full Golgi, single confocal images from WT, mdx, and GFP-tubb6-overexpressing FDB fibers were stained for GM130 and Sec31, GLUT4, or p58. Confocal images were collected. They were opened in Photoshop and GM130 puncta were annotated as associated with the 2nd marker or not. The table shows the numbers of puncta counted*.

### Parallels Between Fiber Cortex Microtubules and Fiber Core SR

**Figure 9:**
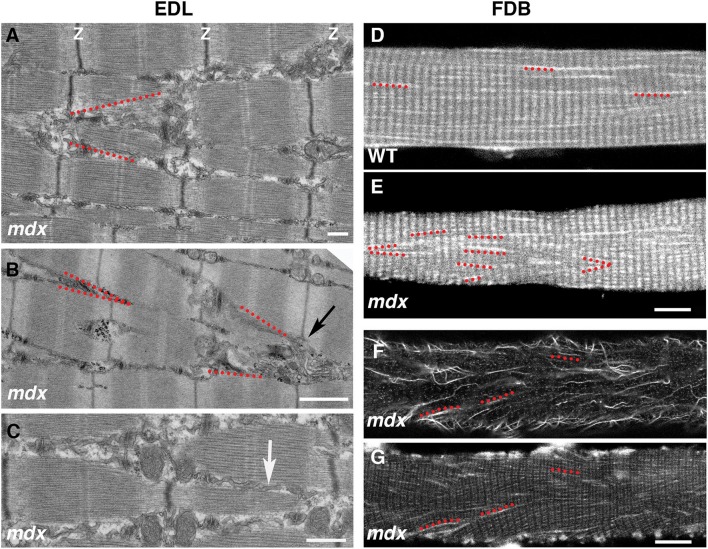
Link between microtubule orientation in the fiber cortex and sarcoplasmic reticulum (SR) orientation in the fiber core. The grid organization is typical of cortical microtubules and GE, likely because of their links to the costameres which only extend through the cortical layer. In the fiber core, longitudinal, and transverse microtubules are in different planes (not shown). However, the angular distribution of microtubules in the *mdx* fiber cortex mirrors that of the SR in the fiber core. On the left side we show the SR organization in electron micrographs of 5 mo-old *mdx extensor digitorum longus* (EDL) muscle. The myofibrillar organization is relatively normal (see thin dark band of Z-disks), despite interruptions by strands of longitudinal SR (best shown in **C**, white arrow) that often form V shapes (red dotted lines in **A,B**). Similar V shapes are found in autofluorescence images of *mdx* FDB fibers, but not of WT ones **(D,E)**. They are also found in tubulin-stained *mdx* FDB fibers **(F)** that were also stained for calsequestrin, an SR marker **(G)**. Bars: **(A)** 500 nm; **(B)** 1 μm; **(C)** 500 nm; **(D–G)** 10 μm.

The cortical microtubule grid differs in its organization from the core microtubules, generally longitudinal without a transverse component. However, we often encounter cortical microtubule bundles, in *mdx* fibers, that form a 20–40 deg angle with the longitudinal fiber axis (see for example box 2 in Figure 5). We were curious about this recurrent orientation and expected to observe it in Second Harmonic Generation (SHG) images of muscle fibers. SHG visualizes muscle myosin heavy chain without need for staining and since SHG is visualized on a confocal microscope it would be simple to determine at what depth such angles form. However, this was not the case. We found an explanation in electron micrographs of muscle fibers of the *extensor digitorum longus* (EDL) *mdx* mouse muscle (Figures 9A–C). Although the Z disks, I and A bands are reasonably well-aligned in these fibers, they are interrupted by cytoplasmic incursions at an angle of ~20 deg. that mostly contain longitudinal SR tubules (Figure 9C, arrow). In fibers that are richer in mitochondria these are also present and we encountered a GE at the origin of such channels (Figure 9B). Similar angular incursions can be seen in autofluorescence images of *mdx* (Figure 9D) but not of WT fibers (Figure 9E). Finally, a microtubule image (Figure 9F) collected at the core-cortex contact of a fiber stained for calsequestrin, an SR marker (Figure 9G), show similar interruptions of the normal pattern. There is therefore a link between microtubules, GE, and SR positioning that extends from cortex to core of the fibers and is affected by the absence of dystrophin, possibly contributing to the weakness of the dystrophic muscle and to structural changes throughout the fiber (Friedrich et al., 2010).

## Discussion

Until we observed the dynamics of WT microtubule and Golgi markers in live muscle fibers (Oddoux et al., 2013), we had no comprehension of their organization. Was this network stable or dynamic? How did GE happen to be positioned at the intersections of a microtubule grid? We answered several basic questions and, as is generally the case, came up with new questions. Here we start investigating the disorganization of the GE-microtubule network in the muscle of the *mdx* mouse in search of answers.

The main new question was: is it possible that dystrophin guides microtubules and anchors GE at the same time? The results presented here show that dystrophin is not necessary to anchor GE but that GE positioning is abnormal in the absence of dystrophin. Thus, dystrophin may play a role, possibly indirect, in positioning GE. To the best of our knowledge, there is no known direct interaction between GE and dystrophin although costameres contain numerous other proteins (Ervasti, 2003). We propose that GE are anchored by ERES, which are membrane domains of the ER. Muscle fibers have a core ER, the sarcoplamic reticulum (SR). The SR is compartmentalized into domains involved in calcium handling and muscle contraction and into domains involved in protein secretion. ERES are part of the latter (Kaisto and Metsikkö, 2003). Interestingly, a subsarcolemmal tubular system shaped like the ER was observed after labeling of skinned muscle fibers with fluorescent dyes (Jayasinghe et al., 2013). We do not know whether the periodic organization of the SR is perturbed in *mdx* fibers but electron microscopy of *mdx* muscle fibers reveals channels that contain SR and cross myofibrils at an abnormal angle of similar magnitude to that often seen in *mdx* microtubules (Figure 9).

We do not know why GE and ERES are increased in number and volume in *mdx* and tubb6 OE fibers. These changes suggest variations in protein secretion. Autophagy defects have been noticed in DMD and *mdx* mice and proposed as a target for DMD treatment (De Palma et al., 2012), so far without success. It is conceivable that Golgi membranes, which serve as source of membrane for autophagosomes (Geng and Klionsky, 2010) are upregulated in an attempt by muscle fibers to compensate for deficient autophagy.

Other organelles may be involved in muscle GE positioning. In WT muscle fibers, GE are often found in pairs (Percival et al., 2010). Interestingly, we found out that these pairs bookend or are bookended by lysosomes (Fukuda et al., 2006). Lysosomes are held by stable, detyrosinated microtubules (Mohan et al., 2019) but this work concerns epithelial cells, not muscle. The role of microtubule stabilization in *mdx* and DMD pathologies is not clear. An increase in microtubule detyrosination has been proposed to make *mdx* microtubules more rigid (Kerr et al., 2015) but there is no consensus that there is a specific increase in detyrosination in *mdx* since its detyrosination level is increased proportionately to an increase in α-tubulin expression (Belanto et al., 2016; Loehr et al., 2018; Randazzo et al., 2019). We only find a minority of muscle microtubules to be detyrosinated (Randazzo et al., 2019 and Figure 4A) but a redistribution of stable microtubules that could affect various organelles cannot be ruled out.

GE pairs and rows are associated with microtubule bundles (Figure 5). This makes sense since muscle microtubules grow bidirectionally along pre-existing microtubules (Oddoux et al., 2013). It is likely that the more GE are positioned in a row, the higher the chance that their microtubules will interact and bundle, perhaps as a reinforcement of microtubule strength and for their protection against severing enzymes (Burkart and Dixit, 2019; Kuo et al., 2019).

It will be interesting to find out more about muscle GC nucleation; so far it has been studied mostly in non-muscle cells. Hot spots and periodic inactivation have been reported (Sanders and Kaverina, 2015) that could contribute to the stochastic character of nucleation from GE. Muscle GE are small however, barely the size of the GC hot spots. Nucleating proteins at non-muscle GC are the same as those involved in centrosomal nucleation (γ-tubulin and the γ-TuRC protein complexes) but with different scaffolding proteins to link them to the GC (Sanders et al., 2017). We have been unable to detect γ-tubulin at muscle GE (Oddoux et al., 2013) but γ-tubulin is even difficult to detect in myotubes because its level is low (Bugnard et al., 2005). The current consensus implicates AKAP450, CDK5Rap2, myomegalin, pericentrin, and centrosomin in the scaffolding of the nucleating machinery at the GC (Sanders and Kaverina, 2015). Pericentrin is the only one of these proteins that we identified in muscle GE; we could not detect AKAP450 (Oddoux et al., 2013). Finding good antibodies has been limiting. In contrast there has been recent progress in understanding microtubule nucleation from muscle nuclei, which can be approached in muscle cultures. Microtubules play a role in nuclear positioning and are important for muscle function (Metzger et al., 2012). The long, detyrosinated microtubules linking nuclei in *mdx* fibers (Figure 4) suggest that nuclei- and GE-based muscle microtubules are distinctly regulated. Microtubule stabilization has been considered a source of pathology in *mdx* (Kerr et al., 2015) but may be a response to the loss of the microtubule grid that normally integrates the nuclei. The central scaffolding protein for nuclei-linked microtubules has been identified as nesprin-1 (Espigat-Georger et al., 2016; Gimpel et al., 2017).

Dystrophin is not the only muscle protein that has been implicated in microtubule guidance; some of these, such as CLIMP-63 (Osseni et al., 2016), a component of the triads, could direct core microtubules and complement dystrophin, CLIMP-63 working in the fiber core, dystrophin in the fiber cortex. Finally, a nNOS splice variant located in muscle GE has also been implicated in microtubule maintenance (Percival et al., 2010). Interestingly, GE themselves are abnormal in these muscle fibers, supporting the importance of Golgi integrity.

**Figure 10:**
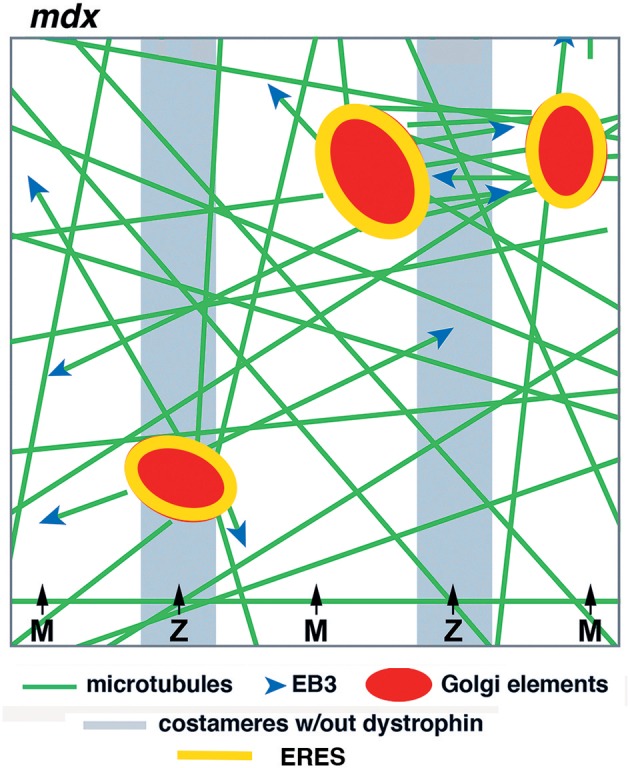
Model of GE and microtubule organization in the cortex of *mdx* muscle fibers. GE and microtubules are disordered. GE are partially aligned with the costameres, microtubules practically not at all in the absence of dystrophin. Dynamic microtubules, with the protein EB3 marking their growing plus-end, are nucleated on the GE but grow in multiple directions, rudderlessly. GE are static and anchored by ERES. Microtubule bundles form between close GE by microtubule-microtubule interactions.

In summary, our work in live fibers of *mdx* and WT mouse muscle has revealed both common features and distinguishing ones that we consider essential. The common features are static GE nucleation and dynamic microtubules coursing along stable microtubules, while essential differences are mispositioning of GE and randomness of newly nucleated microtubules. We hope that the new model (Figure 10), derived from these experiments, will help ask further questions about this fascinating organization and understand it at a molecular and mechanistic level. Microtubules play important roles in in all mammalian cells. It is regrettable that we still understand so little of their organization in the largest of human cells, especially given the likely implication of microtubules in DMD, one of muscle's worst diseases.

## Materials and Methods

### Antibodies

Several antibodies were generous gifts: rabbit anti-sec31/Vp137 from Fred Gorelick and Chris Shugrue (Yale University, New Haven, CT); rabbit anti-p58 ERGIC serum and monoclonal from Jaakko Saraste (University of Bergen, Norway); rabbit anti-GLUT4 (P-1; Ploug et al., 1990) from Thorkil Ploug (University of Copenhagen, Denmark) and rabbit anti-tubb6 (Randazzo et al., 2019) from Jim Ervasti (University of Minnesota-Twin Cities, Minneapolis, MN). Mouse anti-GM130 was purchased from BD Biosciences (Franklin Lake, NJ), rabbit anti-GM130, anti-calsequestrin, and anti-α-tubulin from Abcam (Cambridge, MA), mouse anti-α-tubulin DM1A and rabbit anti-p58 from Sigma-Aldrich (St. Louis, MO), rat anti-α-tubulin from Novus Biologicals (Centennial, CO).

### Plasmids

Tubulin and EB3 plasmids are based on pEGFP-N1 or pEGFP- C1 vectors (Takara Bio Inc., formerly Clontech). p-EB3-GFP-N1 cDNA was a gift from A. Akhmanova (Stepanova et al., 2003). The tubb6 plasmid was generated in the laboratory of Jim Ervasti at the University of Minnesota (Minneapolis, MN). It expresses mouse tubb6 and was built on a pDEST40 vector backbone with N-terminal or C-terminal EGFP tag as fully described in Randazzo et al. (2019).

### cDNA Injection and Electroporation Into Mouse Muscles

All animal protocols were reviewed and approved by the Animal Care and Use Committee of the National Institute of Arthritis and Musculoskeletal and Skin Diseases. Mice were obtained from the Jackson Laboratory (Bar Harbor, ME) and housed and cared for by the personnel of the building 50 Animal Care Facility. Strains used were C57BL/6 or C57BL/10ScSnJ (both WT) and C57BL/10ScSn-DMD^*mdx*^ /J (*mdx*), 6–8 week old unless otherwise mentioned. Since Duchenne Muscular Dystrophy affects males only, all animals were male. Anesthesia with 4% isoflurane and anti-pain injection of 0.05 mg buprenorphine-HCl as well as subsequent steps were as described in Oddoux et al. (2013) and Randazzo et al. (2019). Briefly, hyaluronidase (10 μl of 0.36 mg/ml) was injected through the skin of the heel and left for 1 h. Endotoxin-free plasmid (Genewiz, South Plainfield, NJ) was then injected at 5 mg/ml in sterile Dulbecco's PBS. Electroporation followed, with acupuncture needles as electrodes connected to an ECM 830 BTX electroporator (BTX Harvard Apparatus). Six pulses of 20 ms each at 1 Hz were applied to yield an electric field of 75 V/cm. After 6 days the animals were euthanized, and muscles collected.

### Muscle Fiber Preparation

Briefly, FDB muscles were dissected in sterile PBS, rinsed in sterile DMEM, and incubated for 3 h at 37°C in 1.5 mg/ml type I collagenase from *Clostridium histolyticum* (Sigma-Aldrich) and 1 mg/ml bovine serum albumin in DMEM. Fibers were triturated with 1 ml glass pipets and plated on 22 × 22 mm #1.5 glass coverslips that had been coated for 1 h with a 1:10 dilution of Matrigel (BD Bioscience). Two hours after plating in 0.1 ml of fusion media (DMEM supplemented with 4% horse serum) fibers were fed with the same media supplemented with penicillin-streptomycin (50 U/ml). They were fixed within 24 h to prevent denervation-related reorganization of microtubules and other cytoskeletal components (Ralston et al., 2001, 2006).

### Microtubule Recovery After Nocodazole Treatment

FDB fibers were treated with 4 μg/ml nocodazole for 4 h at 37°C, rinsed three times with fusion medium and left to recover at 37°C for 2, 5, or 15 min. They were then fixed at room temperature with para-formaldehyde (Electron Microscopy Sciences, Atfield PA) diluted to 4% in PBS.

### Fiber Immunofluorescence

Fixed FDB fibers were blocked for 2 h at room temperature in PBS containing 5% bovine serum albumin, 1% normal goat serum, and 0.04% saponin, except for staining with p58 antibodies which needed 0.1% Triton-X100 instead of saponin for permeabilization or −20°C methanol for 20 min for fixation and permeabilization. Fibers were incubated with primary antibodies for 2 h at room temperature (or overnight at 4°C) and with secondary antibodies for 2 h at room temperature, counterstained with Hoechst 33342, and mounted in Vectashield (Vector Laboratories, Burlingame, CA). All washes were 5–15 min at room temperature with PBS containing 0.04% saponin or with PBS alone for samples permeabilized with Triton X-100.

### Light Microscopy Imaging

Images from fixed fibers were acquired on a Leica TCS SP5 with LASAF software or on a Leica TCS SP8 X with LASX software and Lightning, as specified, with 63x N.A. 1.4 Plan Apo objectives. Images were 1,024 or 2,048 pixels wide; pinhole was set to 1.0 Airy Units and channels were scanned sequentially line by line when possible. For Lightning, images were acquired with the same objective, with a pinhole decreased to 0.7–0.5 AU depending on the degree of resolution improvement demanded. Image size was increased in width as set by the LASX. For Z-stacks, system optimized parameters were adopted i.e., optical sections were 0.3 μm-thick for pinhole 1.0, 0.17 μm-thick for pinhole 0.7 and 0.13 μm-thick for pinhole 0.5. 3D image rendition was obtained in the Leica LASX software.

2-Photon excited fluorescence images (2PEF) were obtained with excitation at 920 nm by a 3W MaiTai HP Ti:sapphire photon pulsed laser (Spectra-Physics, Santa Clara, CA) on the Leica SP8 X. Images were collected in a non-descanned detector with a bandpass filter 460/50 nm.

Live fibers were imaged at 0.65 ms/frame in phenol red–free HEPES-supplemented medium (20 mM). Temperature was kept at 37°C, using a Tokai Hit heated stage insert and objective heater. The confocal pinhole was increased up to three Airy Units to increase depth of field and signal intensity while avoiding light damage to the live fibers.

All images were exported as.tif files with lossless compression and assembled into images in Photoshop CC2019 on iMacs working in OS 10.13.6 (High Sierra). Contrast and brightness were adjusted linearly if needed using Levels.

### Image Analysis

As described in Oddoux et al. (2013) EB3-GFP speed was analyzed with PlusTipTracker and PlusTip GetTracks. GFP-tubulin MT growth rate was analyzed with ImageJ (freely available at rsb.info.nih.gov/ij). Kymographs were generated on single or bundled microtubules in ImageJ or FiJi. A rectangle was drawn whose length represents the duration of an event and width the distance covered. Speed was obtained as distance divided by duration, pixels converted into time or length units. Pauses: only the time can be measured. All movies were recorded for 2 mins at 0.65 ms/frame.

Microtubule directionality was measured in the Matlab software TeDT (Liu and Ralston, 2014). The nuclear areas were masked as well as stable MTs remaining after 2 min of nocodazole washout. All the images collected were analyzed. The average directionality curves are calculated from the averages for each fiber, to avoid increasing the weight of better fibers for which more images were obtained than for poorer quality fibers that gave no more than 1 or 2 images suitable for analysis. Some fibers, poorly attached and in a bad shape, were excluded from the study.

For the nocodazole washout experiment, ImageJ was used for all image treatment. For microtubule density quantification, nuclear areas were cropped from the images. For early stage of nocodazole washout (2 min), long nocodazole-resistant microtubules were also cropped from the images to avoid a signal confusing microtubule dynamic recovery. All images were thresholded to make them binary and the number of pixels of microtubules on each image was measured. The total fiber area, minus nuclei, was measured in pixels. For Golgi density quantification, all 415 images were binarized and analyzed in bulk. The ImageJ function “analyze particle” was thresholded at five square pixels to avoid counting the background signal as GE. The size distribution of GE was plotted by binning GE surface by 0.1 μm^2^ steps. The relative frequencies are in % of the total.

Statistical analyses were performed with Prism 8. *T*-tests with Welch's correction were used as most of the data compared did not have the same standard deviation.

3D analysis of confocal Z-stack acquisitions of FDB fibers was performed on Imaris software (version 9.3.1, Bitplane AG, Zurich, Switzerland). 3D reconstruction and surface modeling was used to measure the intensity of Sec31 and GM130 and calculate the volume of GE. Data were exported from Imaris as.xls files and processed on Prism 8 to generate graphs.

### Immunoblots

Immunoblotting was performed as described in Randazzo et al. (2019). Briefly, FDB fibers were plated on Matrigel-coated coverslips. One day after plating, fibers were rinsed once in calcium-free PBS, collected with a rubber spatula and spun down. The pellet was resuspended in 1X sample buffer (National Diagnostic), boiled for 5 min and incubated for 30 min at r.t. Lysates from *Gastrocnemius* were obtained from liquid nitrogen flash-frozen muscles, grounded with mortar and pestle and the powder resuspended in 1X sample buffer (National Diagnostic). Extracts were then boiled for 5 min, incubated at room temperature for 30 min and centrifuged at 14,000 rpm for 10 min. Proteins were separated on 10% NOVEX NuPAGE MES (2-(N-morpholino) ethanesulfonic acid) Bis-Tris (Bis(2-hydroxyethyl)amino-tris(hydroxymethyl)methane) Bis-Tris (Bis(2-hydroxyethyl)amino-tris(hydroxymethyl)methane)1.0 mm gels (Invitrogen) using 1X NuPAGE MES Sodium Dodecyl Sulfate running buffer. The loading amount of each extract was established by first running a gel with the same amount for all extracts and staining the gel with a colloidal blue staining kit (Invitrogen). A 600 dpi image of the gel was obtained with a scanner and the relative loading amount of each extract was calculated by analyzing the intensity of the corresponding lane in ImageJ. Based on the quantitation, a new gel was run under the same conditions. After the run, proteins were transferred to NOVEX nitrocellulose membrane (0.45 μm) in 1X NOVEX NuPAGE transfer buffer (Invitrogen) with 10% methanol for 1 h at 100 V. Blocking of the membranes was performed for 1 h using 5% no-fat milk in Tris-buffered saline solution in presence of Tween-20 (TBS-T: 25 mM Tris, 136 mM NaCl, 3 mM KCl, 0.1% Tween-20, pH 7.4). Primary and horseradish peroxidase-conjugated secondary antibodies were diluted in blocking buffer and incubated for 1 h at room temperature. Washes were performed in TBS-T for three times, 10 min each, after incubations with primary and secondary antibodies. Enhanced Chemiluminescence was performed with SuperSignal West Pico chemiluminescence or SuperSignal Femto Maximum Sensitivity substrates (Thermo Fisher Scientific, Rockford, IL) and peroxidase activity was detected using an XRS^+^ Chemidoc (Bio-Rad, Hercules, CA). Different exposures were captured for each blot and protein bands quantitation was performed using the Image Lab Software v.5.2.1 (Bio-Rad) avoiding pixel saturation on the bands of interest.

### Electron Microscopy

Muscles were fixed in 2% p-formaldehyde/2% glutaraldehyde in 0.1 M sodium cacodylate buffer for 2 h at room temperature and stored at 4°C. Fixed samples were washed in buffer and treated with 1% osmium tetroxide in 0.1 M cacodylate buffer at pH 7.4 for 1 h on ice, washed and en bloc stained with 1% uranyl acetate in 0.1 N acetate buffer at pH 5.0 overnight at 4°C, dehydrated with a series of graded ethanol, and embedded in epoxy resin. Sections 70–90 nm thick were counterstained with uranyl acetate and lead citrate, examined under a JEOL 1200EX transmission electron microscope, and photographed with a bottom-mounted digital CCD camera (AMT XR-100, Danvers, MA, USA). Electron microscopy was carried out at the EM Facility of the National Institute for Neurological Diseases and Stroke.

## Data Availability

The datasets generated for this study are available on request to the corresponding author.

## Ethics Statement

This study was carried out in accordance with the recommendations of the NIAMS Animal Care and Use Committee. The protocol was approved by the NIAMS Animal Care and Use Committee.

## Author Contributions

This work was initiated by SO and KZ and continued and completed by DR, AK, and BA. Each of them performed, to different degrees, the experimental work, collected confocal images, and carried out image analysis. SO also drew the cartoon figures. ER coordinated and led the work, collected images, and drafted the manuscript. All co-authors read and contributed to the manuscript.

### Conflict of Interest Statement

The authors declare that the research was conducted in the absence of any commercial or financial relationships that could be construed as a potential conflict of interest.

## Abbreviations

DMD: Duchenne muscular dystrophy
EDL: *extensor digitorum longus*
ERES: ER exit site
ERGIC: ER-Golgi intermediate compartment
FDB: *flexor digitorum brevis*
fps: frame per second
GE: Golgi Element
GFP: Green fluorescent protein
MTOC: Microtubule organizing center
SR: sarcoplasmic reticulum
TGN: *trans*-Golgi network
WT: wild-type.
