# Supplementary figures and images for: Misplaced Golgi Elements Produce Randomly Oriented Microtubules and Aberrant Cortical Arrays of Microtubules in Dystrophic Skeletal Muscle Fibers
Source: Front Cell Dev Biol. 2019 Sep 18;7:176. doi: 10.3389/fcell.2019.00176 (PMC6759837; doi:10.3389/fcell.2019.00176)

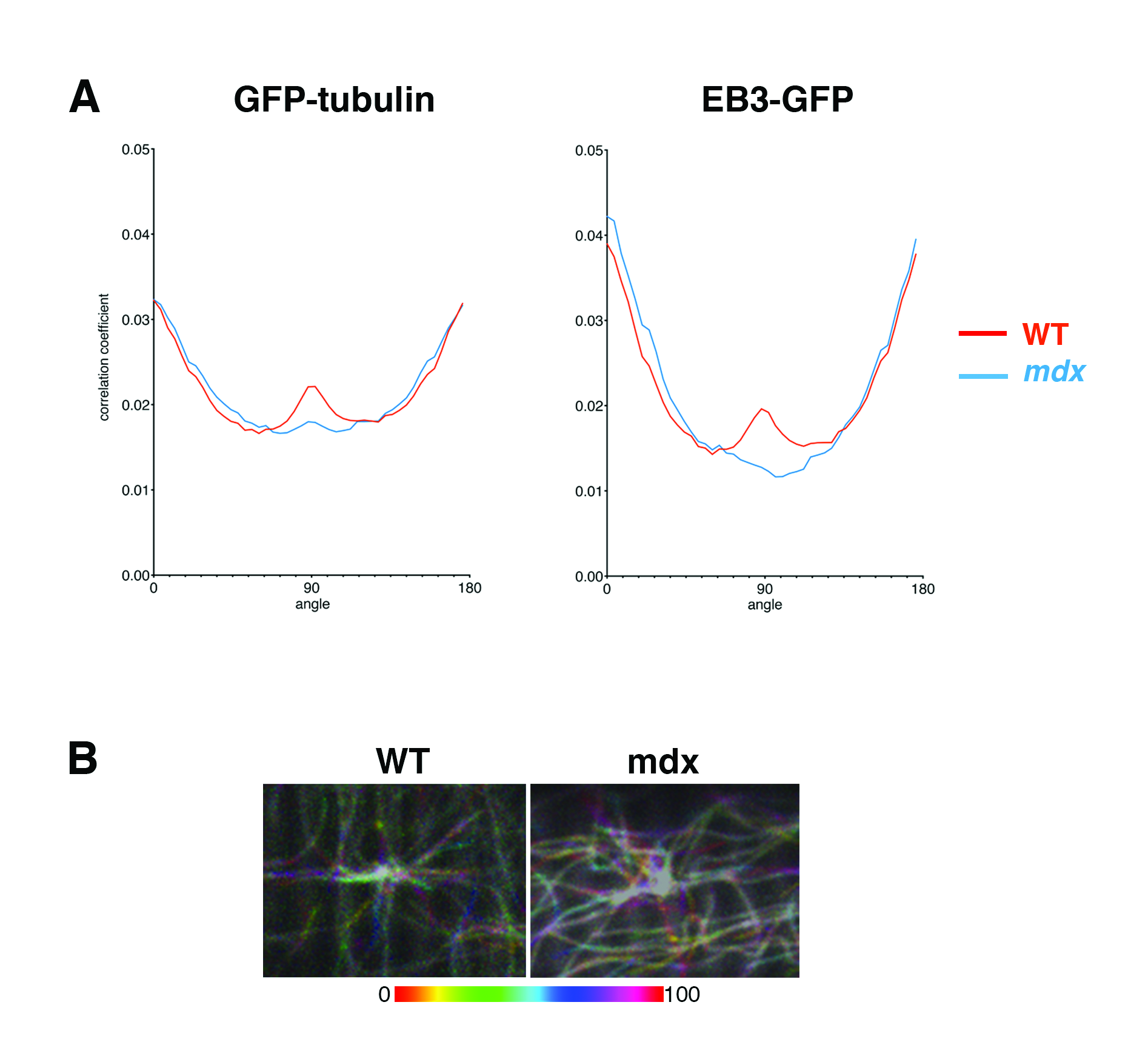

Supplement: Supplementary file 1 [file Image_1.TIF]

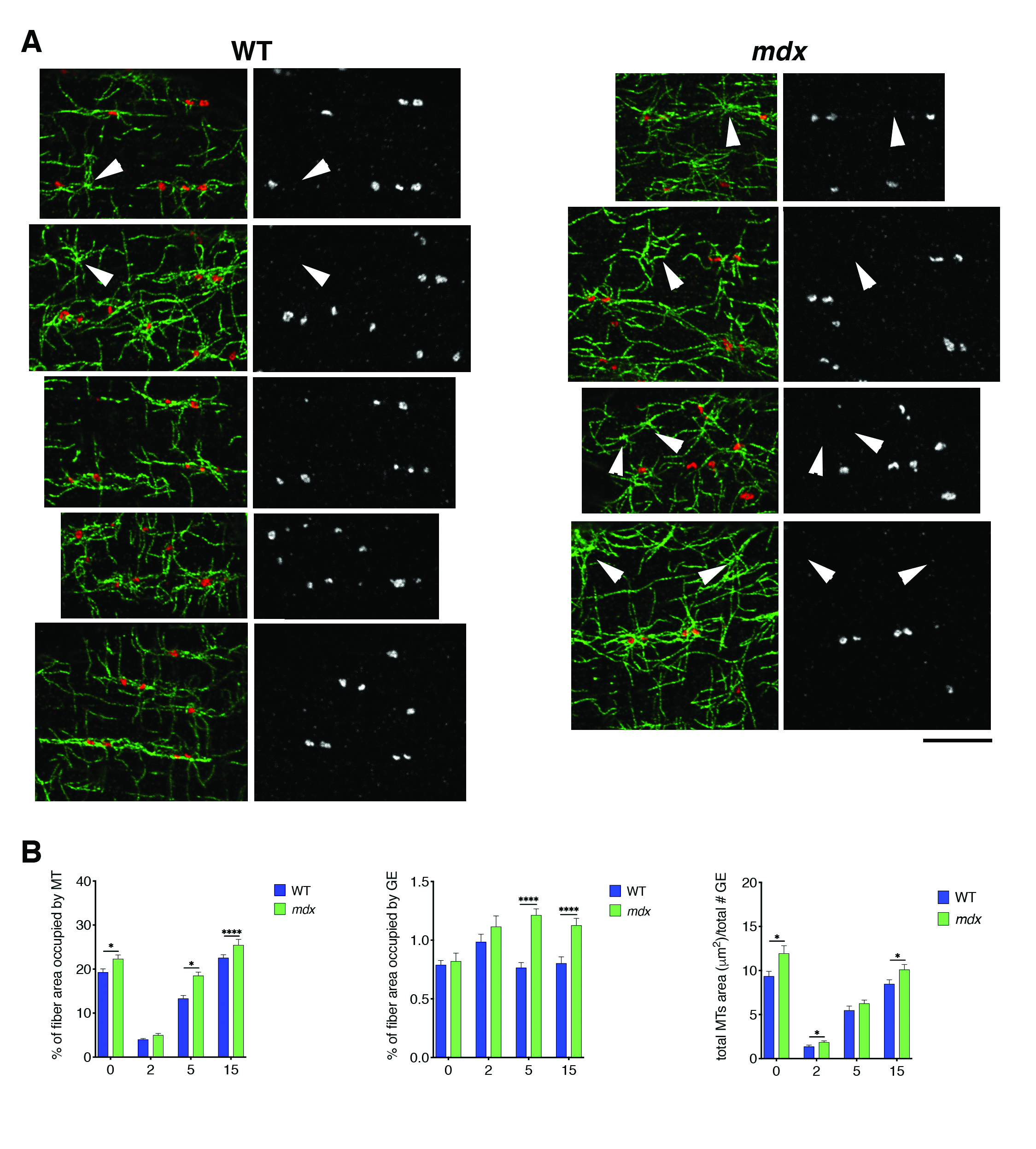

Supplement: Supplementary file 2 [file Image_2.TIF]

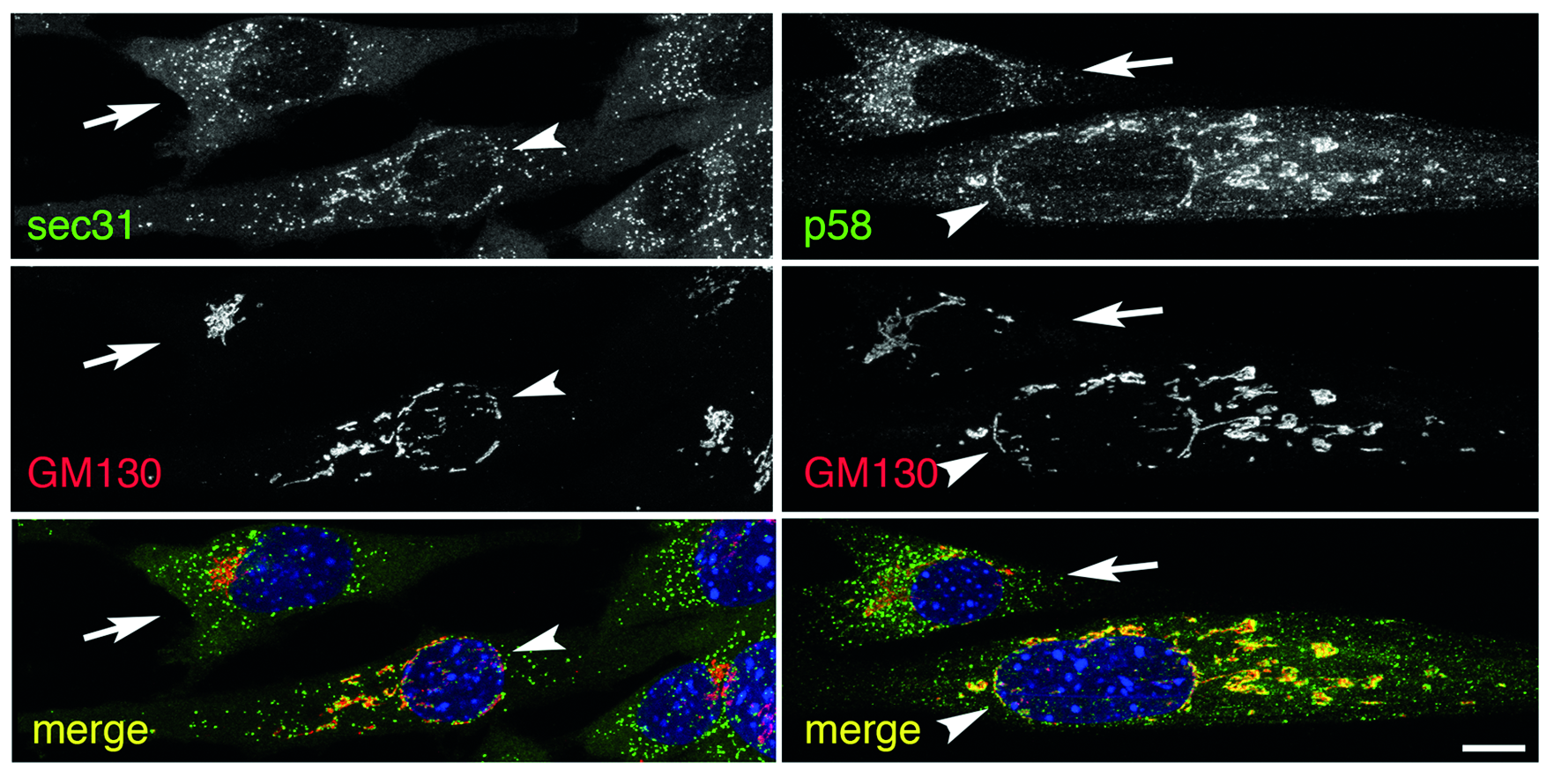

Supplement: Supplementary file 3 [file Image_3.TIF]
