# Supplementary material for: Misplaced Golgi Elements Produce Randomly Oriented Microtubules and Aberrant Cortical Arrays of Microtubules in Dystrophic Skeletal Muscle Fibers
Source: Front Cell Dev Biol. 2019 Sep 18;7:176. doi: 10.3389/fcell.2019.00176 (PMC6759837; doi:10.3389/fcell.2019.00176)
Supplement: Supplementary file 13 [file Data_Sheet_1.DOCX]

**Supplementary Figure Legends**

**Videos 1-7**

Time-lapse recordings were from single fibers of FDB muscles expressing cDNAs encoding GFP-tubulin (Videos 1-5) or EB3-GFP (Videos 6-7). Videos 1-2, 6 were recorded from WT FDB fibers and Videos 3-5, 7 from *mdx* FDB fibers. All time-lapse recordings were acquired at a rate of 93f/min on a TCS SP5 confocal microscope. Fibers were maintained at 37°C (see Material and Methods for additional details).

**Suppl Figure 1**

**A.** The directionality of the microtubules in the projections shown in Fig. 2 was quantitated on thresholded and binarized maximum projections of 2 min recordings. The curves (red for WT; blue: for *mdx*) indicate that the overexpression of the GFP-tagged markers does not alter the original directionality of the microtubule network. **B**. Here we focus on cropped, enlarged fragments of the recordings, showing representative nucleation sites. Projections of 100 frames of GFP-tubulin recordings give in WT an impression of symmetry around the aster-shaped nucleation center. In comparison, the *mdx* example shows a confusing pattern. The live recordings (Videos 8 for WT and 9 for *mdx*) were acquired at a rate of 93f/min on a TCS SP5 confocal microscope equipped with a heated stage and an objective heater at 37°C (see Material and Methods for details). They show that microtubule growth is stochastic in both cases but there is more than one nucleation origin in the *mdx* sample.

**Suppl Figure 2**

**A**. This figure complements Fig. 3 and shows several additional representative fields from WT and *mdx* samples during recovery from treatment with nocodazole. All images showed here were collected after 5 min recovery. *Mdx* fibers present more frequent cases of asters or groups of microtubules without GE staining than WT fibers. Colored panels show GE are in red and microtubules in green. Black and white panels show GE only. **B.** Graphs show that during recovery from nocodazole, *mdx* fibers have denser microtubules and more or larger GE than WT fibers. The increase in microtubule surface per GE is only slightly increased. Bar: 10 μm.

**Suppl Figure 3**

At an early stage of differentiation of C2 muscle cultures, undifferentiated myoblasts (arrows) and differentiated myocytes (arrowheads) can be seen in the same field. Myoblasts have a single GC near the nucleus (GM130 staining, red) but ERES (Sec31staining, green) are spread over most of the cell. Myocytes show a reorganization and convergence of both components; the single Golgi complex turns into several smaller GE while ERES aggregate or fuse into a larger entity. The same situation is observed in cultures stained for GM130 and for p58, a marker of the ER-Golgi Intermediate Compartment (ERGIC). Differences in nuclear size and in shape and size of the myocyte Golgi Elements (GE) result from variability between the cells which are not synchronized. Bar: 10 μm.
